# *Helicobacter pylori* Vacuolating Cytotoxin A: Structure, Biological Functions, Genetic Polymorphisms, and Therapeutic Perspectives

**DOI:** 10.3390/biom16071068

**Published:** 2026-07-22

**Authors:** Xiaona Song, Xiaoqiong Tang, Alfred Tay, Mohammed Benghezal, Barry J. Marshall, Hong Tang, Hong Li

**Affiliations:** 1 Division of Infectious Diseases, State Key Laboratory of Biotherapy and Center of Infectious Diseases, West China Hospital of Sichuan University, Chengdu 610041, China; 2024224060047@stu.scu.edu.cn; 2 Center of Infectious Diseases, West China Hospital, Sichuan University, Chengdu 610041, China; tangxiaoqiong8523@wchscu.cn (X.T.); mbenghezal@gmail.com (M.B.); 3 Laboratory of Infectious and Liver Diseases, Institute of Infectious Diseases, West China Hospital of Sichuan University, Chengdu 610041, China; 4 *Helicobacter pylori* Research Laboratory, Marshall Centre for Infectious Disease Research and Training, School of Biomedical Sciences, University of Western Australia, Nedlands, WA 6009, Australia; alfred.tay@uwa.edu.au (A.T.); barry.marshall@uwa.edu.au (B.J.M.)

**Keywords:** *Helicobacter pylori* infection, vacuolating toxin A, oligomerization, biological function, genotype

## Abstract

VacA (vacuolating cytotoxin A) is a key virulence factor in *Helicobacter pylori* infection, contributing to chronic gastritis and gastric adenocarcinoma. It induces vacuolation, disrupts cellular functions, and modulates immune responses, aiding bacterial survival in the harsh gastric environment. Genetic diversity in the *vacA* gene, particularly alleles like s1 and m1, is associated with more severe clinical outcomes. Recent advances in structural biology, especially cryo-electron microscopy, have revealed VacA’s oligomeric structure and its ability to form anion-selective channels in host cell membranes, providing important insights into its cytotoxic mechanisms. Understanding VacA’s structure and function is essential for unraveling its role in immune evasion and cellular damage. These findings also pave the way for targeted therapeutic strategies, such as subunit vaccines designed to neutralize VacA’s immunosuppressive effects, potentially leading to more effective control of *H. pylori* infections.

## 1. Introduction

*Helicobacter pylori* infection affects nearly 40% of the global population and is typically acquired in childhood. This infection often persists for decades in the absence of effective antibiotic treatment [1,2]. Chronic infection results in active gastritis in virtually all colonized individuals and is a significant risk factor for the development of gastric adenocarcinoma [3].

Among the key virulence factors of *H. pylori*, vacuolating cytotoxin A (VacA) is particularly noteworthy due to its critical role in pathogenesis [4]. The VacA toxin is characterized by its capacity to induce vacuolation in host cells, disrupt cellular functions, and modulate immune responses, thereby facilitating bacterial survival within the harsh gastric environment [5]. The virulence of VacA is significantly influenced by genetic diversity within the *vacA* gene, with specific alleles determining the potency of the toxin and its impact on clinical outcomes [6]. Notably, subtypes such as s1 and m1 are closely associated with severe diseases, including peptic ulcers and gastric cancer, underscoring the relevance of genetic variation in influencing disease severity [7]. Furthermore, several studies have reported that VacA and cytotoxin-associated gene A (CagA), another important virulence factor, exhibit antagonistically interactions in vitro, which may facilitate *H. pylori*’s persistence within the gastric mucosa and contribute to disease progression [8,9,10].

Recent advances in structural biology, particularly through cryo-electron microscopy, have yielded critical insights into the oligomeric structure of VacA and its mechanisms for membrane integration into host cells [11,12,13]. These discoveries are essential for understanding how VacA interacts with host receptors and disrupts cellular homeostasis. This review aims to provide a comprehensive summary of the current knowledge regarding the structure, biological functions, and genetic polymorphisms of VacA, emphasizing its integral role in the pathogenesis of *H. pylori*.

## 2. VacA Structural Characteristics

### 2.1. VacA Synthesis and Protein Domain

*H. pylori* VacA toxin is initially synthesized as a ~140 kDa precursor protein containing all functional domains along with an N-terminal signal sequence [14]. VacA production is tightly regulated at transcriptional, post-transcriptional, and protein-trafficking levels in response to host and culture conditions. For instance, iron limitation upregulates *vacA* transcription [15]; however, conflicting evidence shows reduced VacA abundance in outer membrane vesicles under low iron conditions, suggesting additional regulation at protein trafficking or stability [16]. Moreover, acidic pH downregulates *vacA* transcription but simultaneously stabilizes a conserved 5′ UTR stem-loop, increasing mRNA half-life and sustaining protein levels during acid stress [17]. Similarly, high salt, a dietary risk factor for gastric cancer, upregulates *vacA* transcription (RNA-seq and qRT-PCR) and elevates secreted VacA levels, implying that salt-rich diets may enhance virulence [18]. Notably, mRNA stability serves as a central regulatory node, as the 5′ UTR stem-loop integrates pH and salt signals to maintain VacA production [17]. Although the *vacA* promoter and transcriptional start site are well characterized, the 5′ UTR is key for mRNA stability and protein output [17]. Furthermore, strain-specific variations (s, i, m regions) affect basal toxin activity, yet environmental factors—iron, pH, and salt—modulate expression even within the same strain [17,18]. Overall, these mechanisms ensure preferential VacA production under gastric-like conditions (low iron, acidic pH, high salt), optimizing virulence in the pathogenic niche.

Following ribosomal synthesis in the bacterial cytoplasm, the precursor undergoes post-translational processing that includes: (1) Sec-dependent cleavage of the signal sequence and (2) C-terminal proteolytic processing to yield the mature ~88 kDa toxin [19]. While this maturation process is known to involve specific proteases, their identities remain to be fully characterized [20]. The mature 88 kDa toxin is subsequently secreted via an autotransporter mechanism (type V secretion system, T5SS) [21].

Notably, VacA oligomers can assemble directly from p88 components without requiring proteolysis into p33 and p55 subunits [22]. These subunits exhibit distinct functional specializations: the p33 domain mediates intracellular activities including pore formation and cytotoxicity, while the p55 domain facilitates membrane binding and extracellular interactions [22,23].

### 2.2. VacA Oligomerization

One of the notable features of VacA is its capacity to oligomerize into large multimeric complexes, forming anion-selective channels in host cell membranes, including both cytoplasmic and organelle membranes [24]. In recent years, researchers have employed cryo-electron microscopy (cryo-EM) to resolve the high-resolution three-dimensional structures of VacA oligomers [11,12]. These studies have identified the presence of hexamers and heptamers within a single chiral layer, as well as dodecamers, tridecamers, and tetradecamers, which result from the back-to-back stacking of two chiral layers in solution. Among these oligomers, hexamers and dodecamers are the most predominant, constituting approximately 77% of the sample, while heptamers and tetradecamers constitute the remaining 23% [11].

**Figure 1:**
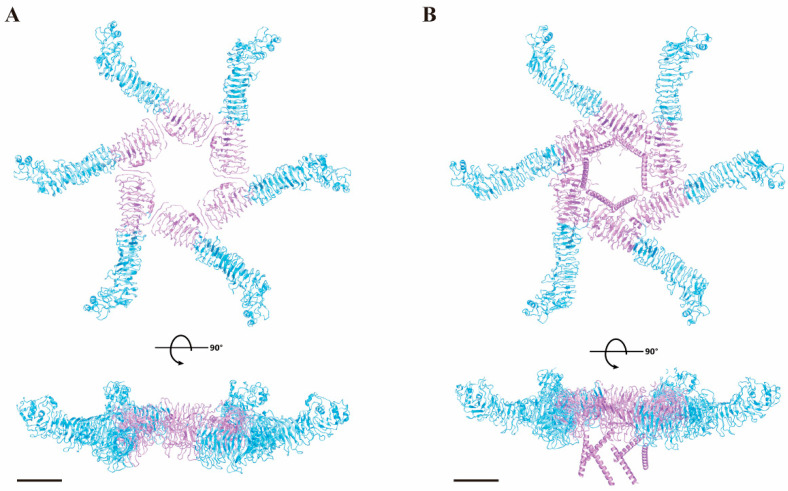
Schematic representation of soluble and membrane-associated VacA hexamer structures. (**A**) Soluble/prepore-like VacA hexamer, illustrated based on the cryo-EM structure of *Helicobacter pylori* VacA hexamer (PDB ID: 6ODY; EMDB ID: EMD-20024) [11]. (**B**) Membrane-associated VacA hexamer, illustrated based on published structural analyses of membrane-associated or membrane-inserted VacA hexamers [13,14]. The (**top**) panels show en face views, whereas the (**bottom**) panels show views rotated 90° around the *x*-axis. The p33 domain is shown in violet, and the p55 domain is shown in light blue. Scale bar, 50 Å. The pore-forming region is shown schematically and should not be interpreted as a resolved structure of the mature functional VacA channel.

To elucidate the structural details of VacA, the hexameric structure with the highest resolution was analyzed in detail. The VacA hexamer is formed by the interaction of six identical monomers. Each monomer consists of an N-terminal p33 domain and a C-terminal p55 domain, which are connected by a flexible linker region that allows for significant conformational changes between the two domains during oligomerization. The monomers are arranged around a central pore, creating a highly symmetrical hexameric structure that resembles a wheel or ring (Figure 1A) [12].

Upon binding to lipid membranes, VacA oligomers undergo distinct conformational rearrangements. Cryo-EM analyses have revealed that, following membrane association, the central region of VacA oligomers displays a pronounced N-terminal α-helical density, which contrasts with the lack of central density observed in VacA oligomers formed in solution [14]. The α-helices of VacA extend from each protomer, indicating a critical pore-forming region essential for its cytotoxic function. Some oligomers fully integrate into the lipid bilayer, while others either partially embed or remain atop the membrane surface, highlighting structural heterogeneity (Figure 1B). The p55 domain exhibits notable flexibility, facilitating dynamic interactions with the lipid bilayer. This structural adaptation is pivotal for VacA to assemble functional anion-selective channels, thereby contributing to toxin-mediated cellular damage [12,13].

### 2.3. VacA Membrane Channel Formation

The p55 domain anchors VacA to the cell surface by recognizing and binding to specific receptors on the cell membrane, such as sphingolipids and receptor-like protein tyrosine phosphatase (RPTP) [25,26,27]. The p55 domain is predominantly localized at the channel periphery, where it stabilizes the interaction between the toxin and the cell membrane, maintaining the structural integrity and functionality of the channel [28]. Following receptor binding, the p33 subunit embeds into the membrane through its hydrophobic regions, forming a cylindrical channel that spans the lipid bilayer [12,13,14]. Quantitative structural characterization has provided detailed insights into the VacA channel architecture. The cryo-EM has determined the structure of the VacA hexamer at 3.8 Å resolution, revealing that each protomer (p88) consists predominantly of a continuous right-handed β-helix extending from the p55 domain into the p33 domain [11,14]. The functional membrane-inserted form assembles as a hexamer with a molecular weight of approximately 0.528 MDa [11]. Atomic force microscopy (AFM) imaging of membrane-associated VacA two-dimensional crystals has further defined the overall dimensions: the hexamer exhibits an overall diameter of ~28 nm and protrudes ~2.9 nm from the bilayer surface, with a maximal height of ~8 nm when including the membrane-spanning portion [29]. While the precise pore diameter remains to be determined experimentally due to the conformational flexibility of the pore-forming N-terminus, these structural parameters provide a quantitative framework for understanding the channel’s organization. The GXXXG motif within the p33 domain-comprising two glycine residues separated by three variable amino acids- plays a pivotal role in VacA pore channel formation [13,24,30,31]. Structural analysis, particularly via crystallography and cryo-EM, demonstrate that this motif is strategically positioned to mediate helix-helix interactions critical for assembling a stable, cylindrical pore. Site-directed mutagenesis disrupting the GXXXG motif markedly impairs the toxin’s channel-forming ability [13,31]. Furthermore, the conservation of this motif across various strains of VacA highlights its importance in mediating the toxin’s pathogenic effects on host cells [14,31,32].

Although it is well established that VacA oligomerizes (primarily into hexamers) on membranes and forms functional pores by inserting its hydrophobic N-terminal region (p33 domain) into the lipid bilayer, the precise timing of membrane insertion and its relationship with oligomerization remain debated. Some studies suggest that pre-formed hexamers (a “pre-pore” state) remain partially dissociated until membrane contact triggers a conformational change in the N-terminal hydrophobic region (residues 30–37), causing it to extend from the oligomeric core into the membrane to form a pore [12,13]. In contrast, other evidence indicates that soluble oligomers (e.g., hexamers) dissociate into monomers before the p33 domain inserts into the lipid bilayer and reassembles into a functional hexameric channel [14]. Furthermore, oligomerization and membrane insertion are not strictly coupled, as certain oligomerization-deficient mutants (e.g., VacA Δ346–347) retain membrane insertion capability [14]. Resolving these discrepancies will require additional high-resolution structural and functional studies to clarify the spatiotemporal dynamics of VacA pore formation and the contributions of individual domains.

The channels exhibit anion selectivity, with a pronounced preference for chloride ions, which plays a critical role in disrupting cellular ionic homeostasis [33]. Quantitative characterization of the channel properties from planar lipid bilayer recordings has established a permeability sequence of Cl^−^ ≈ HCO_3_^−^ > pyruvate^−^ > gluconate^−^ ≫ K^+^, with a permeability ratio of PCl^−^/PNa^+^ ≈ 4.2:1, indicating moderate anion over cation selectivity [34,35]. Single-channel conductance measurements revealed a unitary conductance of approximately 24 pS in symmetric 1.5 M NaCl solutions, with values ranging from 10 to 30 pS depending on ionic conditions [35,36]. The halide permeability sequence (SCN^−^ >> I^−^ > Br^−^ > Cl^−^ > F^−^) suggests that anion discrimination is governed by a “weak-field-strength” binding site, a feature shared with certain ligand-gated anion channels [35]. This disturbance alters key cellular process, including signaling pathways, membrane potential, and survival mechanisms. Chloride influx through VacA channels induces osmotic imbalance, resulting in the swelling of endosomal compartments and the formation of large vacuoles, which is a hallmark of VacA intoxication (discussed below) [14]. Beyond their role as passive ion conduits, these channels also facilitate urea diffusion across epithelial membranes, further contributing to their pathogenic effects. The combined disruption of ionic homeostasis and secondary osmotic effects underscores VacA’s central role in *H. pylori* pathogenesis [36].

The channel-forming activity of VacA is strictly regulated by both acidic pH and the chemical composition and properties of the target membrane. Acidic pH (below 5.0) serves as an essential switch that triggers a conformational transition: at neutral pH, VacA exists predominantly as soluble dodecamers (two hexamers assembled face-to-face), whereas upon acidification, these dodecamers disassemble and reassemble into membrane-inserted hexameric pores [29]. This pH-dependent rearrangement is a prerequisite for the toxin’s vacuolating cytotoxicity. In parallel, the lipid composition of the target bilayer plays an equally critical role: pore formation strictly requires the presence of anionic phospholipids (e.g., phosphatidylserine), as membranes composed exclusively of zwitterionic lipids fail to support VacA binding or insertion regardless of pH, underscoring the essential role of electrostatic interactions between the toxin and negatively charged lipid headgroups [29]. Membrane fluidity further modulates the insertion efficiency of the hydrophobic N-terminal region (residues 1–26), as tightly packed or highly ordered lipid bilayers impede the conformational rearrangements necessary for pore assembly [37]. Additionally, cholesterol- and sphingolipid-enriched lipid raft microdomains provide a favorable platform for VacA oligomerization and channel stabilization, while membrane curvature and lateral tension may fine-tune the conformation and gating properties of the inserted channel [37]. Collectively, these observations establish that VacA channel formation and function are precisely governed by the lipid composition and biophysical state of the target membrane, ensuring that toxin activity is selectively deployed in appropriate membrane environments. Notably, neither acidic pH alone nor anionic lipids alone are sufficient; both conditions must be met simultaneously, confirming that VacA activity is precisely tuned to the acidic pH and specific ionic/lipid environment characteristic of the gastric mucosa.

## 3. VacA–Host Cell Interactions for *H. pylori* Persistence and Pathogenicity

**Figure 2:**
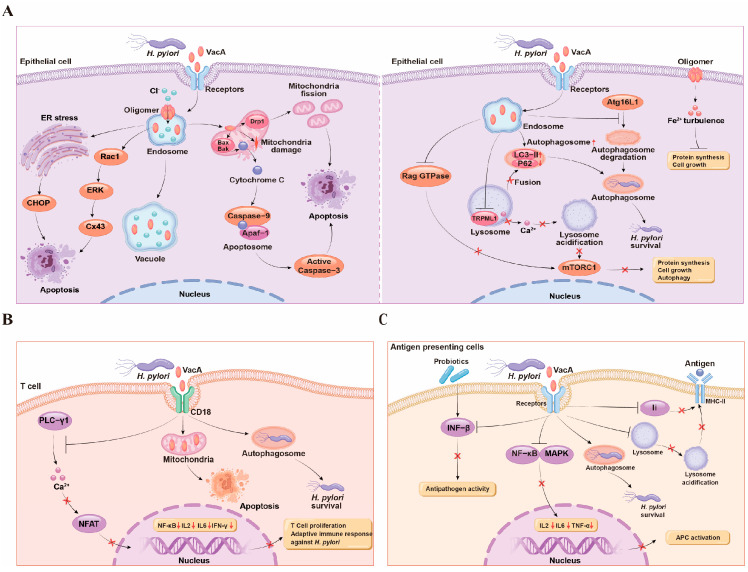
The interplay between VacA and host epithelial cells, as well as immune cells, modulates multiple host responses. (**A**) *H. pylori* that are attached to or swimming within the mucus can secrete VacA into the surrounding environment. VacA is a pore-forming toxin capable of binding to various host receptors on epithelial cells, such as sphingomyelin, RPTP, phospholipids, LRP1, and others. Once internalized into epithelial cells, VacA induces the formation of large vacuoles by traveling ion channels on the endosome. Additionally, VacA can induce mitochondria and endoplasmic reticulum-associated apoptosis, autophagosome formation, and interfere with cell metabolism. (**B**) VacA can bind with CD18 on T-cells, inhibiting the NFAT signaling pathway, which results in the blockade of T cell proliferation and adaptive immune response. VacA can also induce mitochondrial-associated apoptosis and autophagosome formation. (**C**) VacA can bind to host receptors on antigen presenting cells, including sphingomyelin, phospholipids and RPTP, which induces autophagosome formation, and prevents lysosome acidification-associated antigen recognition, immune inflammatory response, as well as probiotic-mediated antipathogen activity. Black arrows represent activated signaling pathways, while T-bars indicate inhibitory pathways. The “×” symbol indicates the termination of the signaling pathways. Red up arrows indicate an increase in expression, while red down arrows indicate a decrease in expression. RPTP, receptor type protein-tyrosine phosphatases; LRP1, low-density lipoprotein receptor-related protein 1; ER, endoplasmic reticulum; Rac1, Ras-associated C3 botulinum toxin substrate 1; ERK, extracellular regulated protein kinases; CHOP, C/EBP homologous protein; Drp1, dynamin-related protein 1; Apaf1, apoptotic protease activating factor 1; Cx43, connexin 43; LC3-II, microtubule-associated protein 1 light chain 3 beta II subunit; P62, sequestosome 1; TRPML1, transient receptor potential subfamily m member 1; Atg16L1, autophagy related protein 16 like protein 1; mTORC1, mammalian target of rapamycin complex 1; CD18, integrin member β2; PLC-γ, phospholipase c gamma 1; NFAT, nuclear factor of activated T cells; INF-β, interferon beta; NF-κB, nuclear factor-kappaB; MAPK, mitogen-activated protein kinase; Ii, invariant chain; TNF-α, tumour necrosis factor alpha; MHC, major histocompatibility complex.

The interaction of VacA with host epithelial cells and immune system cells is a complex and multifaceted process that plays a critical role in the bacterium’s ability to manipulate host cell functions and evade immune responses (Figure 2) [38].

### 3.1. Targeting Epithelial Cells

#### 3.1.1. VacA Induces Vacuolation in Epithelial Cells

VacA derives its name from its ability to induce large cytoplasmic vacuoles in epithelial cells, a phenotype that becomes evident within hours of exposure and is exacerbated by weak bases [39]. Quantitative assessment of VacA cytotoxicity has been performed primarily using neutral red uptake assays in cultured epithelial cell lines. In human gastric epithelial AZ-521 cells, purified VacA at a concentration of 120 nM induced a time-dependent decrease in intracellular ATP levels with reduction of approximately 20% at 6 h, 35% at 12 h, and 50% at 24 h [40]. Mitochondrial membrane potential, assessed by flow cytometry, declined from 52 ± 3 to 24 ± 1 within 6 h of VacA treatment [40]. In HeLa cells, typical vacuolation assays employ VacA concentrations of 5–10 µg/mL to assess cytotoxic activity [41]. In vivo quantitative assessment of VacA cytotoxicity has been achieved using a chronic intragastric infusion model in mice. Infusion of 500 nM VacA for 3 or 30 days induced parietal cell vacuolation in gastric tissue, with vacuolation scores ranging from 0 to 4 (corresponding to 0% to >75% of parietal cells affected), depending on exposure duration [42]. This approach provides a quantitative framework for evaluating VacA cytotoxicity within the physiological gastric environment.

The current model of VacA-induced vacuolation involves multiple steps: (1) binding to cell surface receptors such as sphingomyelin, RPTP, phospholipids, and low-density lipoprotein receptor-related protein 1 (LRP1), followed by endocytic uptake [25,27,43]; (2) trafficking to late endosomes and lysosomes [44]; and (3) formation of anion-selective channels in endosomal membranes, leading to chloride ion (Cl^−^) and water accumulation that drives vacuole formation [33,45]. The accumulation of VacA not only directly affects ion flow through these channels but also amplifies its effects by modulating key signaling pathways [44]. By disrupting the late stages of the endocytic pathway, VacA impairs the normal functions of endosomes and lysosomes, hindering their degradation and recycling processes [33,45]. This results in macromolecular accumulation and broader cellular homeostasis dysregulation. Studies implicate dynamin and syntaxin 7 in VacA-mediated vacuolation, though their precise roles remain unclear [46,47]. By inhibiting protein degradation and disrupting cellular signaling, VacA causes profound cellular dysfunction that may culminate in apoptosis or other forms of cell death (Figure 2A) [48].

#### 3.1.2. VacA Induces Apoptosis in Epithelial Cells

A central pathogenic effect of VacA on epithelial cells is the induction of apoptosis. The toxin’s N-terminal region targets the inner mitochondrial membrane, forming ion channels that disrupt mitochondrial dysfunction [30,49]. This triggers cytochrome c release into the cytosol, initiating apoptosis [30,50,51]. VacA further amplifies this process by activating pro-apoptotic proteins Bax and Bak, which promote additional cytochrome c release [52]. Notably, VacA-induced apoptosis depends on its channel-forming ability but operates independently of conventional extrinsic mitochondrial apoptotic pathways [30,50].

VacA also engages mitochondrial fission machinery through activation of dynamin-related protein 1 (Drp1), inducing excessive mitochondrial fragmentation that exacerbates cell death and inflammation [51]. Additionally, VacA promotes Bax accumulation on endosomes, facilitating endosome-mitochondria juxtaposition to accelerate apoptosis [48]. Emerging evidence suggests endoplasmic reticulum (ER) stress and C/EBP homologous protein (CHOP) contribute to VacA-induced apoptosis, though further in vivo validation is required [53].

Beyond mitochondrial targeting, VacA dysregulates autophagy by activating the Rac1-ERK pathway, leading to aberrant connexin 43 (Cx43) accumulation in autophagic vesicles [54]. This impaired autophagy, coupled with Cx43 dysregulation, represents another mechanism through which VacA induces apoptosis [54]. The ability of VacA to induce cell death via multiple pathways underscores its central role in *H. pylori* pathogenesis (Figure 2A).

#### 3.1.3. VacA’s Regulation of Autophagy in Epithelial Cells

VacA exposure triggers autophagosome formation while blocking their maturation, resulting in accumulation of dysfunctional autophagosomes that may provide a protective niche for *H. pylori* survival [55]. Mechanistically, VacA engages LRP1 to upregulate LC3-II while downregulating p62, thereby stimulating autophagosome biogenesis [55]. However, VacA interferes with the autophagosome-lysosome fusion and degradation processes via LRP1, resulting in a sustained elevation of autophagic flux [43]. Furthermore, VacA promotes *H. pylori* infection in the host by interfering with the Atg16L1-mediated autophagic pathway, which is essential for maintaining cellular homeostasis and immune defense. Variants of *atg16L1* that impair autophagy have been shown to increase susceptibility to *H. pylori* infection in humans, underscoring the importance of this pathway in pathogen clearance [56]. VacA exploits this vulnerability by disrupting autophagosome formation and maturation, thereby inhibiting the degradation of intracellular bacteria [56]. Recent studies have demonstrated that VacA can inhibit the function of transient receptor potential channel mucolipin 1 (TRPML1), preventing the effective release of calcium ions from the lysosome into the cytoplasm [57]. By inhibiting this process, VacA disrupts lysosomal acidification. Insufficient acidification of the lysosome hinders the fusion of autophagosomes with lysosomes, leading to ineffective degradation of *H. pylori* within autophagosomes and facilitating bacterial survival within host cells (Figure 2A) [57,58].

#### 3.1.4. The Impact of VacA on Epithelial Cell Metabolism

Studies have shown that VacA disrupts the intracellular and extracellular ion balance by forming anion-selective channels on the epithelial cell membrane [24,50]. In addition, VacA profoundly disrupts intracellular iron transport pathway by binding to lipid raft structures on epithelial cell membranes, such as sphingolipid or LRP1. This interaction alters the storage locations and utilization efficiency of iron, resulting in abnormal iron distribution both within and outside the cell [59]. A recent study has also shown that VacA can inhibit the mechanistic target of rapamycin complex 1 (mTORC1) pathway, blocking cell survival signals and affecting normal cellular metabolism and viability [60]. mTORC1 is a crucial signaling complex that regulates cell growth, proliferation, and metabolism, playing a central role in sensing signals related to nutrients, energy status, and oxygen levels. Under normal conditions, the activation of the mTORC1 signaling pathway promotes protein synthesis, cell proliferation, and the inhibition of autophagy [60]. However, VacA disrupts the amino acid sensing mechanism, preventing effective transport of amino acids to the lysosome, which in turn inhibits the activation of upstream regulators such as Rag GTPase. Consequently, due to this suppression of amino acid sensing, mTORC1 cannot be effectively activated [60]. Additionally, VacA impedes lysosomal acidification by inhibiting the calcium channel TRPML1, further obstructing the effective activation of mTORC1 (Figure 2A) [57,60].

### 3.2. Interaction with Immune System Cells

#### 3.2.1. Inhibition of VacA on T Cells

VacA can bind to integrin subunits, particularly CD18, which pairs with CD11 to form the β2 integrin complex and is primarily expressed on T leukocytes [61]. Upon binding to CD18, VacA effectively invades the primary human T-cells, which was regulated by protein kinase C (PKC) isoforms PKCη and PKCζ through phosphorylation of threonine residue T758 in the cytoplasmic tail of CD18 [62]. After CD18-dependent entry into cultured human T cells, VacA disrupts the calcium ion flux necessary for T cell activation by inhibiting the phospholipase C-γ1 (PLC-γ1) pathway [61]. This inhibition results in the suppression of nuclear factor of activated T cells (NFAT) activation, leading to a significant decrease in the transcription of genes essential for T cell proliferation and cytokine production, such as those involved in the NF-κB pathway and encoding IL-2, IL-6, and IFN-γ [63,64]. Consequently, the impaired activation and reduced cytokine secretion weaken the host’s adaptive immune response against *H. pylori* (Figure 2B).

In addition, VacA induces T cell apoptosis via a mitochondrial-dependent pathway. It targets the mitochondrial membrane, causing disruption of the mitochondrial transmembrane potential and the release of cytochrome c into the cytosol [49]. This process is facilitated by the activation of pro-apoptotic proteins Bax and Bak, which form pores in the mitochondrial membrane [43,52]. The release of cytochrome c subsequently activates caspases, leading to cell death. This apoptotic pathway effectively reduces the number of active T cells, further compromising the host’s immune defense and promoting bacterial persistence. Similar to its effects in epithelial cells, VacA induces the formation of autophagosomes in T cells; however, it prevents their maturation and fusion with lysosomes, resulting in a cellular environment characterized by the accumulation of undegraded autophagosomes T cells (Figure 2B) [43,53,55].

#### 3.2.2. VacA’s Inhibition of Antigen Presentation

Studies have demonstrated that VacA can adversely affect the function of APCs, thereby disrupting the host immune response (Figure 2C). Firstly, VacA inhibits the activation of macrophages and dendritic cells, significantly reducing the secretion of pro-inflammatory cytokines such as IL-2, IL-6, and TNF-α. This reduction directly diminishes the host immune system’s ability to recognize and respond to antigens [64,65]. Additionally, VacA impairs antigen processing by disrupting lysosomal acidification in macrophages, which in turn hampers lysosomal degradation. This interference with the endocytic pathway directly reduces the effective processing and presentation of antigens, weakening the immune functions of APCs [66]. VacA has also been demonstrated to impair antigen presentation processes in B lymphocytes by interfering with the invariant chain (Ii)-dependent pathway, especially the chain (Ii)-dependent proteolytic processing of tetanus toxoid, and inhibiting the expression of integrin-linked kinase (ILK). These effects collectively hinder antigen presentation mediated by newly synthesized major histocompatibility complex (MHC) class II molecules [67,68]. Furthermore, VacA has been shown to reduce IFN-β production in response to probiotics such as *Lactobacillus acidophilus*, thereby attenuating the activation of antiviral and antibacterial immune responses [66]. Similar to its effects on epithelial cells, VacA exposure also induces autophagy while simultaneously inhibiting autophagosome maturation, resulting in a cellular environment characterized by the accumulation of undegraded autophagosomes in APCs [55,58]. These mechanisms indicate that VacA not only suppresses the host immune response by directly interfering with antigen presentation but also weakens overall immune defenses by inhibiting key immune signaling pathways, allowing *H. pylori* to persist and evade clearance within the host.

Due to its capacity to impair T-cell function and antigen presentation, VacA has emerged as a promising candidate for vaccine development. Detoxified VacA, whether chemically inactivated with formaldehyde or produced as a recombinant protein in *Escherichia coli*, retains its antigenicity while exhibiting partial immunogenicity [69,70]. Preclinical studies in animal models have shown that VacA-based vaccines, when administered with adjuvants, confer significant protection against *H. pylori* infection [71,72]. In clinical trials, a multi-epitope vaccine incorporating recombinant VacA, CagA, and neutrophil-activating protein (NAP) demonstrated excellent safety profiles, durable immune responses, and robust antigen-specific T-cell activation [73]. These findings highlight VacA as a strong candidate for inclusion in future *H. pylori* vaccine formulations, though further research is needed to optimize its immunogenicity, delivery strategies, and long-term protective efficacy.

## 4. VacA Genetic Polymorphisms

### 4.1. Allelic Variations in vacA Gene and Their Impact on H. pylori Virulence

**Figure 3:**
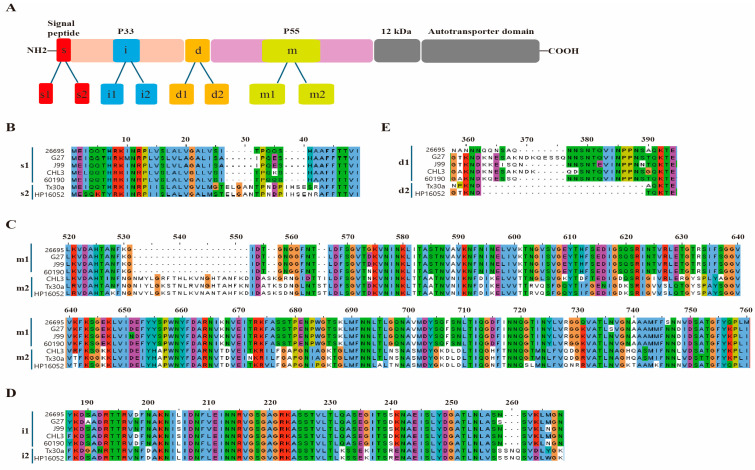
VacA organization and allelic diversity. (**A**) The organization of the 140 kDa VacA with significant allelic diversity in the signal region (s1 and s2), intermediate region (i1 and i2), d-region (d1 and d2), and the mid-region (m1 and m2); (**B**) Representative sequences of the s- region of type s1 (found in strains 26695, G27, J99, CHL3 and 60190) and type s2 (found in strains Tx30a and HP16052) VacA proteins are shown. Secreted type s2 VacA proteins contain an amino-terminal extension relative to the secreted type s1 VacA proteins; (**C**) Representative m-region sequences of type m1 and m2 VacA; (**D**) Representative sequences of i-region sequences for type i1 and type i2 VacA; (**E**) Representative d-region sequences for type d1 and type d2 VacA. The VacA protein sequences of the indicated *H. pylori* strains were retrieved from the National Center for Biotechnology Information (NCBI) database. In panel (**A**), different colors are used to distinguish the different polymorphic regions of the VacA protein. In panels (**B**–**E**), amino acid residues are colored according to their physicochemical properties using the Clustal color scheme implemented in Jalview.

Studies have demonstrated that vacA gene exhibits allelic variation across four distinct regions, leading to the production of VacA proteins with differing levels of cytotoxicity (Figure 3A) [6]. The first region of variability, known as the s-region, is located in the signal peptide and the N-terminal portion of the p33 subunit, with two main variants identified as s1 and s2 (Figure 3B). The s1 variant encodes a VacA protein capable of forming membrane channels at a significantly higher rate and inducing vacuole formation in host cells, whereas the s2 variant produces a form of the toxin that forms membrane channels slowly and lacks this vacuolating ability [74].

The second region of *vacA* diversity is found within the p55 subunit, which has two primary variants: m1 and m2 (Figure 3C). The m1 variant is characterized by a higher affinity for cell binding and enhanced channel-forming activity, resulting in increased cytotoxicity and more severe cellular damage [75]. In contrast, the m2 variant exhibits significantly reduced cell-binding capacity and channel-forming efficiency, leading to a lower overall cytotoxicity [76]. Strains harboring the m2 variant are often associated with milder clinical outcomes, such as chronic gastritis, and are less frequently linked to severe gastroduodenal diseases [7].

The third region of variability, referred to as the i-region, is also observed in the p33 subunit, but is located closer to the C-terminus. Polymorphisms within this region mainly give rise to 3 alleles, i1, i2, and i3 (predominant i1 and i2) (Figure 3D) [77]. The functional differences between these subtypes are thought to arise from variations in their vacuolating cytotoxin activity and T-cell immune function inhibition [77,78,79]. The i1 subtype, similar to the s1/m1 genotype, is associated with higher virulence and a greater likelihood of severe clinical outcomes, including gastric cancer [80]. This association has been particularly well-documented in populations from East Asia, where the i1 subtype is prevalent in *H. pylori* strains isolated from patients with advanced gastric diseases. Conversely, the i2 subtype is generally linked to a lower risk of severe disease, mirroring the clinical patterns observed with the s2/m2 genotype [81].

The fourth region of variability is termed the d-region, comprising the d1 and d2 variants, which are located at the junction of the p33 and p55 subunits (Figure 3E). The *vacA* d2 genotype contains a deletion of 23 to 27 amino acids between the *vacA* i and m regions, whereas the d1 genotype does not have this deletion [82]. Epidemiology studies have shown that the d1 subtype of the VacA is closely associated with the development of severe gastrointestinal diseases, such as gastric ulcers and gastric cancer [82,83]. In contrast, the d2 subtype of the VacA protein demonstrates significantly reduced activity in these pathogenic mechanisms [82,83]. In addition to the well-characterized s-, i-, m-, and d-regions, other polymorphic regions of *vacA* have also been described. The c-region, defined by a 15 bp deletion at the 3′ end of the p55 domain and classified as c1 or c2, has been associated with gastric cancer risk in several studies [83,84]. More recently, Xue et al. reported that the c1 genotype was significantly associated with gastric cancer and remained significant in multivariate analysis [85]. Moreover, a C-terminal “n-region” or “tail-region” within the p55 domain has been identified and classified into n1 and n2 genotypes based on a 2-amino-acid deletion, although its functional and clinical significance remains less well established [86].

Overall, the genetic diversity of *vacA* gene gives rise to multiple genotype combinations, including but not limited to s1/m1/i1/d1, s1/m1/i2/d1, s1/m2/i1/d2, s1/m2/i2/d2, s2/m1/i1/d1, s2/m1/i2/d1, s2/m2/i1/d1, and s2/m2/i2/d2 [7,85]. These combinations exhibit varying levels of cytotoxicity and are closely associated with different clinical manifestations of disease [84,87]. Among these, the s1/m1/i1/d1 genotype is identified as the most virulent combination, often linked to severe clinical outcomes such as gastric ulcers and gastric cancer [88]. This genotype enhances the toxin’s ability to bind to host cell membranes, thereby increasing its activity and inducing more pronounced cellular damage and apoptosis. In contrast, the s2/m2/i2/d2 genotype represents a low-toxicity combination associated with milder gastrointestinal diseases, such as chronic gastritis, and is less frequently connected with severe disease outcomes [6,89].

### 4.2. Geographical Distribution of VacA Genotypes

The genetic diversity of the *vacA* gene, particularly in the s- and m-regions, exhibits significant geographical variation (Table 1), which is closely associated with regional differences in clinical outcomes and disease risk associated with *H. pylori* infection [3]. For instance, the less toxic s2/m2 VacA type is more frequently identified in strains from regions such as the North America, Egypt and Western Europe, which are associated with a lower risk of gastric cancer [90,91,92]. In contrast, the highly toxic s1/m1 VacA type is more prevalent in *H. pylori* strains from East Asia, South America, and regions where there is high incidence of gastric cancer [93,94].

### 4.3. Interplay Between VacA and CagA Polymorphisms

The clinical significance of VacA polymorphisms is further amplified by their interaction with other *H. pylori* virulence factors, particularly CagA (an oncoprotein in *H. pylori*). The co-occurrence of specific VacA and CagA significantly enhances the pathogenic potential of *H. pylori*, contributing to more severe disease outcomes [91,93]. For instance, it was reported that the presence of the s1/m1 VacA genotype in conjunction with CagA-positive strains is a strong predictor of progression from chronic gastritis to pre-neoplastic lesions and ultimately to gastric cancer [93]. Interestingly, one observational study suggests an association between *H. pylori cagA*/*vacA*-s1/m1 and the risk of developing substantial or advanced fibrosis in metabolic dysfunction-associated steatotic liver disease (MASLD), which requires validation through further large-scale clinical studies and research into the underlying biological mechanisms [139].

These co-occurrence effects may be attributed to the antagonistic interactions of VacA and CagA on host cellular pathways. VacA suppresses NFAT activation induced by CagA, balancing inflammatory responses and preventing excessive host cell damage [9]. Furthermore, CagA could interfere with VacA-induced apoptosis to inhibit the turnover of gastric epithelial cells, promoting long-term bacterial colonization [8,10]. These regulatory interactions may ensure the persistence of *H. pylori* within the gastric mucosa and contribute to its pathogenicity. Currently, however, only a limited number of studies have thoroughly examined the functional interaction of CagA and VacA polymorphisms on disease outcomes. Further in-depth research is essential to unravel the functional interactions between VacA and CagA genetic variations, which could offer valuable insights into the pathogenic mechanisms underlying the clinical diversity of *H. pylori* infections.

## 5. Drugs and Vaccine Development Targeting *H. pylori* VacA

To date, no specific drugs directly targeting *H. pylori* VacA have been licensed for clinical use, and no VacA-based vaccine is currently available. Current clinical management of *H. pylori* infection relies on antibiotic-based eradication regimens (e.g., amoxicillin, tetracycline, metronidazole) combined with proton pump inhibitors and bismuth salts, or vonoprazan combined with high-dose amoxicillin, whose effects remain attributable to bacterial clearance rather than specific VacA inhibition [1,140]. Nevertheless, VacA has emerged as an active focus of drug discovery efforts, with several experimental strategies under investigation. Small-molecule inhibitors have been explored through computational screening. In one study, FDA-approved antibiotics were repurposed and five candidates including cefiderocol, cefpiramide, doxycycline, enoxacin, and tedizolid were identified as possessing stable binding affinity to VacA, among which cefiderocol exhibited the most potent predicted inhibitory activity (MM-PBSA: −28.33 kcal/mol) [141]. Another virtual screening campaign using the ZINC15 database identified seven lead compounds with favorable binding affinity and pharmacokinetic profiles [142]. Aurora kinase A inhibitor MLN8054 was shown to reverse VacA-induced mitochondrial fragmentation and restore gastric epithelial integrity in human antrum gastric organoids and mouse models [49]. In addition, the NF-κB inhibitor BAY11-7082 attenuated VacA-mediated apoptosis and inflammatory damage by modulating the TRAF1/4-1BB/NF-κB axis [143]. A distinct approach involves guide peptides targeting VacA, generated by phage display and fused to antimicrobial peptides (e.g., pexiganan), which enhanced specific toxicity against *H. pylori* by 64- to >256-fold compared with off-target bacteria [144].

All VacA-based vaccine candidates remain at preclinical or early-stage clinical investigation, with none having progressed beyond Phase 2 trials [145,146]. Successful toxin-targeting vaccines have been established against other bacterial pathogens, such as tetanus and diphtheria toxoids [147]. For *H. pylori* VacA, however, this therapeutic approach has not yet been achieved. The failure of VacA-based vaccines to achieve clinical translation stems from three interconnected obstacles. First, VacA exhibits substantial allelic diversity and functional pleiotropy, including pore formation, immunosuppression, and pro-apoptotic activity, which complicates the identification of universally protective epitopes and creates uncertainty regarding which functional domain should be neutralized [148]. Second, effective immunity against gastric *H. pylori* requires mucosal responses (sIgA and tissue-resident T cells), yet conventional parenteral routes are inadequate for this purpose, while mucosal delivery faces degradation, mucus entrapment, and tolerogenic gastrointestinal microenvironment [146,149]. Third, although VacA has been implicated in colonization in some experimental models [150], it is primarily recognized as a cytotoxin and virulence determinant. Therefore, neutralizing VacA alone would be expected mainly to attenuate toxin-mediated epithelial injury and immune modulation, rather than directly block the major colonization mechanisms of *H. pylori*, such as acid acclimation and bacterial adherence, which are largely mediated by urease activity and multiple adhesins [151]. These theoretical limitations are corroborated by a pivotal Phase 1/2 human challenge trial in which a VacA/CagA/NAP combination vaccine, despite being immunogenic, failed to provide significant protection against experimental challenge, confirming that targeting secreted virulence factors alone is insufficient for prophylaxis and necessitating the inclusion of colonization factors in future vaccine designs [152].

VacA belongs to the diverse superfamily of bacterial pore-forming toxins (PFTs), which compromise host cell membrane integrity yet display substantial differences in structure, activation, and receptor recognition. Major PFT families include β-PFTs, which form membrane-spanning β-barrels (e.g., cholesterol-dependent cytolysins and hemolysins), and α-PFTs, which utilize α-helices for pore formation (e.g., RTX toxins and colicins) [153]. Among β-PFTs, cholesterol-dependent cytolysins (CDCs) are secreted by Gram-positive pathogens, including *Streptococcus pneumoniae* pneumolysin (PLY), *Listeria monocytogenes* listeriolysin O (LLO), and *Streptococcus pyogenes* streptolysin O (SLO) [154]. These toxins are structurally conserved and typically comprise four domains. Domain 4 (D4) mediates membrane recognition through cholesterol-interacting loops and a conserved undecapeptide motif that contributes to toxin activation and conformational rearrangement [154,155]. This cholesterol-dependent membrane targeting contrasts with VacA, which preferentially associates with anionic phospholipids and undergoes activation under acidic conditions [29]. Following membrane binding, CDCs oligomerize into large ring- or arc-shaped prepore complexes composed of approximately 34–50 subunits, which are substantially larger than the hexameric channels formed by VacA [29,154]. Despite these mechanistic differences, both toxin families exploit lipid microdomains to facilitate membrane insertion: CDCs primarily use cholesterol-rich membrane platforms, whereas VacA associates with lipid rafts [156]. In addition, both toxins can exert pore-independent effects that contribute to immune modulation and bacterial persistence [155,157].

The RTX (Repeats in ToXin) family, produced by Gram-negative pathogens such as uropathogenic *Escherichia coli* α-hemolysin (HlyA) and *Bordetella pertussis* adenylate cyclase toxin (CyaA), is characterized by glycine- and aspartate-rich nonapeptide repeats, commonly represented as GGXGXDXUX, that coordinate calcium ions [158,159]. RTX toxins generally require post-translational acylation for full activation. Unlike VacA, which forms anion-selective channels through a prepore assembly pathway, many RTX toxins generate small, transient, cation-selective pores through a growing-pore mechanism [29,35,159]. Nevertheless, VacA and certain RTX toxins share the ability to affect intracellular targets. VacA can localize to mitochondria and promote apoptosis, whereas some RTX-family toxins, such as *Vibrio cholerae* RtxA/MARTX toxin, disrupt the actin cytoskeleton by catalyzing actin cross-linking, thereby inducing cell rounding [30,160].

The biomedical potential of bacterial PFTs is well exemplified by CDCs, whose detoxified forms (PLY, LLO, SLO) have been incorporated into vaccines, including pneumococcal formulations and the Combo4 candidate against *S. pyogenes* [161,162]. Beyond immunization, CDCs serve as targeting ligands for drug delivery and as components of responsive nanocarriers. Meanwhile, anti-toxin approaches, such as nanoparticle-based sequestration and small-molecule inhibitors, offer alternatives to conventional antibiotics [163,164]. These successful strategies provide valuable references for VacA-focused development. For instance, classical toxoid vaccines (e.g., tetanus, diphtheria) rely on enzymatic specificity and antigenic conservation to enable detoxification without epitope loss. More creatively, certain toxins have been repurposed as delivery tools: CTB and LTB function as mucosal adjuvants and antigen carriers, overcoming a major hurdle in *H. pylori* vaccination [165]; ClyA has been adapted as an OMV-based display platform for heterologous antigens; and liposomal decoy nanoparticles sequester PFTs in vivo as an anti-virulence measure that circumvents antibiotic resistance. Together, these toxinological paradigms, ranging from neutralization to functional repurposing, offer a coherent conceptual foundation for guiding future VacA-targeted interventions [166].

## 6. Conclusions and Future Perspectives

In summary, VacA represents a quintessential multifunctional bacterial toxin that orchestrates key aspects of *H. pylori* pathogenesis. By interacting with diverse host cell receptors and modulating critical cellular pathways, VacA plays a central role in gastric disease progression. The clinical relevance of VacA allelic variation underscores the need for further investigation into strain-specific contributions to disease outcomes.

Understanding VacA’s molecular mechanisms and its role in *H. pylori* persistence remains a key research focus. Structural characterization of VacA, particularly through cryo-EM, is critical for: (1) elucidating the precise pathogenic mechanisms of VacA-mediated immune evasion and cellular disruption, and (2) enabling the rational design of detoxified VacA-based vaccine components. These structural insights will be crucial for developing targeted strategies to neutralize VacA’s immunosuppressive properties, essential for effective *H. pylori* vaccines. Combined with functional studies of VacA’s interactions with host cells, these advances will provide a foundation for novel therapeutic interventions against *H. pylori* infection.

## Figures and Tables

**Table 1:** Geographical distribution of *vacA* allelic types of *H. pylori* according to the World Health Organization regions.

WHO Region	Countries Providing Data (No. of Studies)	No. of Isolates	Predominant S-Region (%, Mean Value)	Predominant M-Region (%, Mean Value)	Refs.
Africa region	South Africa (4), Nigerian (1), Morocco (1)	644	s1 (63)	m1 (55)	[89,95,96,97,98]
Americas region	North America: the United States (2), Canada (1)	347	s1 (66)	m2 (56)	[90,99]
	Central America: Costa Rica (2), Mexico (4), Cuba (2)	835	s1 (80)	m1 (70)	[100,101,102,103,104,105,106]
	South America: Brazil (2), Argentina (1), Venezuela (2), Peru (2), Chile (1), Ecuador (1), Colombia (1)	998	s1 (84)	m1 (66)	[76,93,99,101,107,108,109,110,111]
Eastern Mediterranean region	Egypt (4), Tunisia (1), Kuwait (1), Saudi Arabia (1), Iran (3), no specific country (1)	1055	s1 (56)	m2 (59)	[91,99,112,113,114,115,116,117]
European region	Eastern Europe: Czech Republic (1), Hungary (1), Poland(1), Romania (1), Slovenia (1)	365	s1 (78)	m2 (55)	[99,118]
	Western Europe: Germany (2), The Netherland (2), France (1), Italy (1), Sweden (2), Spain (1), Portugal (2), Ireland (1), Belgium (1), England (1)	792	s1 (71)	m2 (56)	[92,99,119,120,121,122,123,124]
Southeast Asia region	Bhutan (1)	209	s1 (100)	m1 (78)	[94]
Western Pacific region	China (8)	1070	s1 (97)	m2 (63)	[85,99,124,125,126,127,128,129,130]
	Japan (4), South Korea (3)	841	s1 (92)	m1 (77)	[99,131,132,133,134,135,136]
	Malaysia (1), Singapore (1), Vietnam (2)	1162	s1 (97)	m1 (56)	[116,137,138]
	Australia (1)	24	s1 (66)	m2 (58)	[99]

## Data Availability

No new data were created or analyzed in this study. Data sharing is not applicable to this article.

## Abbreviations

VacA	vacuolating cytotoxin A	MHC	major histocompatibility complex
*H. pylori*	*Helicobacter pylori*	CD11	cluster of differentiation 11
CagA	cytotoxin-associated gene A protein	PKC	protein kinase C
5′ UTR	5′ untranslated region	IL-2	interleukin-2
RNA-seq	RNA sequencing	IL-6	interleukin-6
qRT-PCR	quantitative reverse transcription polymerase chain reaction	IFN-γ	interferon gamma
T5SS	type V secretion system	APCs	antigen-presenting cells
cryo-EM	cryo-electron microscopy	ILK	integrin-linked kinase
PDB	Protein Data Bank	NAP	neutrophil-activating protein
EMDB	Electron Microscopy Data Bank	NCBI	National Center for Biotechnology Information
RPTP	receptor-type protein tyrosine phosphatase	WHO	World Health Organization
AFM	atomic force microscopy	MASLD	metabolic dysfunction-associated steatotic liver disease
LRP1	low-density lipoprotein receptor-related protein 1	FDA	Food and Drug Administration
CD18	cluster of differentiation 18	MM-PBSA	molecular mechanics/Poisson–Boltzmann surface area
NFAT	nuclear factor of activated T cells	TRAF1	TNF receptor-associated factor 1
ER	endoplasmic reticulum	4-1BB	CD137/TNF receptor superfamily member 9
Rac1	Ras-related C3 botulinum toxin substrate 1	sIgA	secretory immunoglobulin A
ERK	extracellular signal-regulated kinase	PFTs	pore-forming toxins
CHOP	C/EBP homologous protein	RTX	repeats-in-toxin
Drp1	dynamin-related protein 1	CDCs	cholesterol-dependent cytolysins
Apaf1	apoptotic protease activating factor 1	PLY	pneumolysin
Cx43	connexin 43	LLO	listeriolysin O
LC3-II	microtubule-associated protein 1 light chain 3 beta II	SLO	streptolysin O
p62	sequestosome 1	D4	domain 4
TRPML1	transient receptor potential mucolipin 1	HlyA	α-hemolysin
Atg16L1	autophagy-related protein 16-like 1	CyaA	adenylate cyclase toxin
mTORC1	mechanistic target of rapamycin complex 1	MARTX	multifunctional-autoprocessing repeats-in-toxin
PLC-γ1	phospholipase C gamma 1	CTB	cholera toxin B subunit
IFN-β	interferon beta	LTB	heat-labile enterotoxin B subunit
NF-κB	nuclear factor-kappa B	ClyA	cytolysin A
MAPK	mitogen-activated protein kinase	OMVs	outer membrane vesicles
TNF-α	tumor necrosis factor alpha		
